# Talks with Dr. Mandeville

**Published:** 1892-06

**Authors:** 


### HALL'S JOURNAL OF HEALTH

TRUTH DEMANDS NO SACRIFICE; ERROR CAN MAKE NONE.

Vol. 39. JUNE, 1892. No. 6.

### TALKS WITH Dr. MANDEVILLE.

No. 5.

Q. Doctor, will you kindly resume your talk about the nerves?

A. Yes, all ready, sir.

I have taken up so much time on this point because it seems to me an exceedingly important thing to show you that the nerve fibre, the little white thread which carries the message along, is not any thing very different from the things we are accustomed to behold.

In the meantime, let us assume that this operation which is merely the working of a piece of machinery set in motion at one end and continues working towards the other.

I want to make it a little clearer that there is a close analogy between the falling of the cards and what I may call the falling of the successive molecules in the nerves. In ordinary life you speak of things falling into easier positions, when you do not exactly mean things falling towards others. There are many things which are analogous to the nerve structure, one is a train of gunpowder. Gunpowder is composed of various substances for each other's burning, and the gas which is to burn them is obtained not from the air, but from the gunpowder itself. Now, what happens when the gunpowder is burnt? It is simply that the different sorts of molecules fall into the position which they occupied primarily; and you will see that the molecules falling into easier and more natural positions will advance rapidly and set fire to the magazine.

Now, we see that in the gunpowder we have different substances mixed together, but in the explosion of nitro-glycerine something occurs very much like what happens in case of the communicating nerves. The molecules of nitro-glycerine are all alike. When you shake this substance and make it explode, there is no formation of new molecules, but there is just a rearrangement of the molecules, which are disturbed and unable to maintain their former position, thus forming gas.

This gas requires a great deal of room, consequently there is an enormous expansion. This explanation of the transmission of motion in the nerve fibre is given very clearly by Spencer, but the best physical evidence that I know of is contained in certain experiments of DuBois-Raymond. These experiments form a very long story and I do not propose to trouble you with an account of them. It is quite sufficient to assume that the transmission of the message along a nerve is occasioned by the falling into an easier position of the successive molecules of the nerve.

I spoke just now of the nerve as a "string of molecules." It is not exactly a "string." The nerve consists of a sheath containing within it a fatty substance, and inside of that is the "axis cylinder" of the nerve fibre itself, which is an exceedingly minute thing. Five or ten thousand of them, or even more, could lie in the length of an inch. You know that the molecules of water are so small that from 200 to 2,000 millions of them could lie in a centimetre, which is about one-third of an inch, therefore, from 600 to 6,000 millions can lie in the space of an inch. Now, you see that supposing the nerve fibre to be composed of molecules of water, there would be from 120,000 to 1,200,000 of them in an inch's breadth, but the molecules of which the nerve fibre is composed are very much larger than the molecules of water.

This fact being conceded as to the transmission of motion along the nerve fibres, either way, we are brought to another class of nerve fibres, which exist in the body and which apparently contradict the fact of the hot spoon experiment. There are certain nerve fibres whose ends come out in your skin, which are very sensitive, and which can be disturbed by the slightest possible touch. The message passing up your arm along these very delicate white threads, goes up the back of the neck and comes down again ; not upon the same threads, but upon an entirely different series of threads. Those which carry up the message are called *sensory* fibres, because they convey the sensations we feel. They are the nerves used by the senses to carry the message to the brain. The nerves which bring the message back and tell my hand to jump out of the way of the hot spoon, are called *motor* fibres, because they cause my hand to move. The discovery that there were two entirely different sets of nerves in the body, some carrying messages *to* the brain and some *from* it, was that of Sir Charles Bell. You will see that this is apparently a contradiction of the fact I mentioned to you last, viz.: that the nerve which carries the message goes up to the central office, and never comes down from it to the end of the fibre. On the other hand, the message which comes along the motor nerve always travels in *one* direction, it comes *down* from what I call the central office to the end of the fibre, where it is embodied in muscle and makes the muscle move. It has been found that if you excite a sensory nerve at the central office, it will come *down* the other way; again, if you were to get at the head of the motor nerve and excite it at the central office, it would transmit *upward*, but not *downward* upon the opposite sensory nerve.

The reason why messages do not go forward and backward upon the same nerve is not that the nerves are incapable of carrying such messages, but that the ends of them are not so arranged as to deliver them when they are carried, you know that a telegraph message is carried with enormous rapidity; that the delay which takes place in the delivery of the message is caused by the time which it takes to produce the different signs corresponding to the various words or letters, and the time it takes to convey it to the person to whom it is addressed. The actual time it takes to send a signal along the telegraph wire is inconsiderable, whereas the time taken by the transmission of the nerve message is not at all minute, that is to say, in the case of a nerve message in your own bodies the distance being very short, the time is very small, but the velocity is not equal to what we find in other cases, for example, in that of light, which travels nearly 200,000 miles a second, while the nerve message travels only about 90 feet a second.

This question of velocity along the nerve was solved long ago by Professor Helmholtz in an exceedingly interesting way, which I will relate to you on some future occasion.

There is a certain nerve in a frog's leg which moves in a peculiar muscle, and there is an analogous nerve which moves a corresponding muscle in the human body. This nerve in the frog can be cut out, with the muscle still attached to it, and it is found that for a long time both nerve and muscle remain alive, their condition indeed remaining very much the same as when they were in the body. During this time the nerve can be excited by sending the electric fluid along it, and the excitement in the nerve causes the muscle to contract endways and sideways. You can feel this action by taking hold of your biceps and bending your arm. The muscle will then swell up and get shorter, and at the same time it swells out sideways. That is produced by a message coming down your nerve and spreading itself out in innumerable small ramifications into the muscles.

Now, when a message is sent from the extremity of the body up to the central office and back again, the central office we speak of, means a collection of this gray matter in the brain, a collection of houses of cards, so to speak, all put together—are set falling down as soon as this message comes to them, and may in turn set a number of other houses falling down which represent the motor nerves. That is a thing very analogous to what really happens when we send a telegraph message to a small town in the neighborhood of a larger one, the gray matter standing in the place of the central office where the messages are received, and where corresponding messages are sent out.

[To be continued.]

